# Triethyl­ammonium 4-(3,5-dinitro­benzamido)-*N*-(3,5-dinitro­benzo­yl)benzene­sulfonamidate

**DOI:** 10.1107/S1600536812050180

**Published:** 2012-12-15

**Authors:** Ghulam Waris, Humaira Masood Siddiqi, Ulrich Flörke, Rizwan Hussain, M. Saeed Butt

**Affiliations:** a Department of Chemistry, Quaid-I-Azam University, Islamabad 45320, Pakistan; b Universität Paderborn, Warburgerstrasse 100, D-33098 Paderborn, Germany; c NESCOM, PO Box 2216, Islamabad, Pakistan

## Abstract

The mol­ecular structure of the title salt, C_6_H_16_N^+^·C_20_H_11_N_6_O_12_S^−^, shows a planar geometry of the benzamido–phen­yl–sulfonyl moiety, with a dihedral angle of 1.59 (9)° between the aromatic ring planes. The central ring and the aromatic ring of the other dinitro­benzamide group are nearly perpendicular, making a dihedral angle of 89.55 (9)°. All nitro groups lie almost in plane with the associated aromatic rings, the O—N—C—C torsion angles ranging from 9.2 (2) to 24.3 (2)°. In the crystal, strong anion–anion N—H⋯O and anion–cation hydrogen bonds form inversion dimers stacked along the *a* axis. Less prominent anion–anion C—H⋯O inter­actions lead to the formation of a three-dimensional network including anion–anion dimers as well as anion–anion chains along [100?].

## Related literature

For background to polyamide-imide and other high-temperature resistant polymeric materials, see: Kawakami *et al.* (2003 ▶). For the structure of phthalylsulfacetamide, see: Shin *et al.* (1984 ▶).
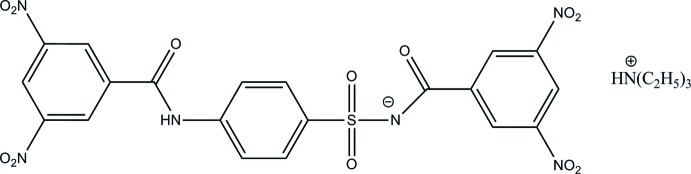


## Experimental

### 

#### Crystal data

C_6_H_16_N^+^·C_20_H_11_N_6_O_12_S^−^
*M* *_r_* = 661.61
Triclinic, 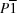

*a* = 9.1046 (12) Å
*b* = 13.2050 (18) Å
*c* = 13.3427 (18) Å
α = 98.854 (3)°
β = 105.147 (3)°
γ = 105.494 (3)°
*V* = 1448.3 (3) Å^3^
*Z* = 2
Mo *K*α radiation
μ = 0.19 mm^−1^
*T* = 130 K
0.38 × 0.37 × 0.19 mm

#### Data collection

Bruker SMART APEX diffractometer
Absorption correction: multi-scan (*SADABS*; Sheldrick, 2004 ▶) *T*_min_ = 0.931, *T*_max_ = 0.965
13859 measured reflections
6875 independent reflections
4652 reflections with *I* > 2σ(*I*)
*R*_int_ = 0.032

#### Refinement

*R*[*F* ^2^ > 2σ(*F* ^2^)] = 0.041
*wR*(*F* ^2^) = 0.084
*S* = 0.89
6875 reflections
415 parameters
H-atom parameters constrained
Δρ_max_ = 0.36 e Å^−3^
Δρ_min_ = −0.36 e Å^−3^

### 

Data collection: *SMART* (Bruker, 2002 ▶); cell refinement: *SAINT* (Bruker, 2002 ▶); data reduction: *SAINT*; program(s) used to solve structure: *SHELXS97* (Sheldrick, 2008 ▶); program(s) used to refine structure: *SHELXL97* (Sheldrick, 2008 ▶); molecular graphics: *SHELXTL* (Sheldrick, 2008 ▶); software used to prepare material for publication: *SHELXTL* and local programs.

## Supplementary Material

Crystal structure: contains datablock(s) I, global. DOI: 10.1107/S1600536812050180/pv2607sup1.cif

Structure factors: contains datablock(s) I. DOI: 10.1107/S1600536812050180/pv2607Isup2.hkl

Supplementary material file. DOI: 10.1107/S1600536812050180/pv2607Isup3.cml

Additional supplementary materials:  crystallographic information; 3D view; checkCIF report

## Figures and Tables

**Table 1:** Hydrogen-bond geometry (Å, °)

*D*—H⋯*A*	*D*—H	H⋯*A*	*D*⋯*A*	*D*—H⋯*A*
N1—H1*A*⋯O8^i^	0.88	2.00	2.8611 (17)	165
N100—H10*P*⋯O2^ii^	0.93	1.86	2.747 (2)	158
C13—H13*A*⋯O8^i^	0.95	2.22	3.136 (2)	162
C3—H3*A*⋯O8^i^	0.95	2.45	3.221 (2)	139
C6—H6*A*⋯O11^iii^	0.95	2.38	3.215 (2)	147
C9—H9*A*⋯O10^iv^	0.95	2.48	3.384 (2)	159
C20—H20*A*⋯O3^v^	0.95	2.31	3.225 (2)	163

Symmetry codes: (i) 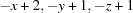; (ii) 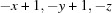; (iii) 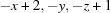; (iv) 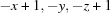; (v) 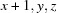.

**Fig. 1.**
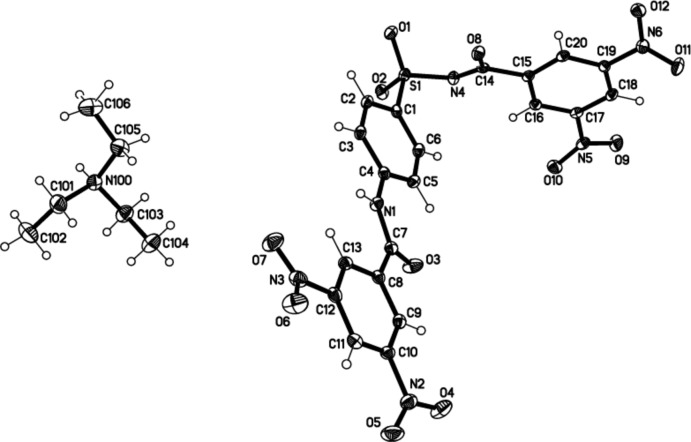
Molecular structure of the title compound with anisotropic displacement parameters drawn at the 50% probability level.

**Fig. 2.**
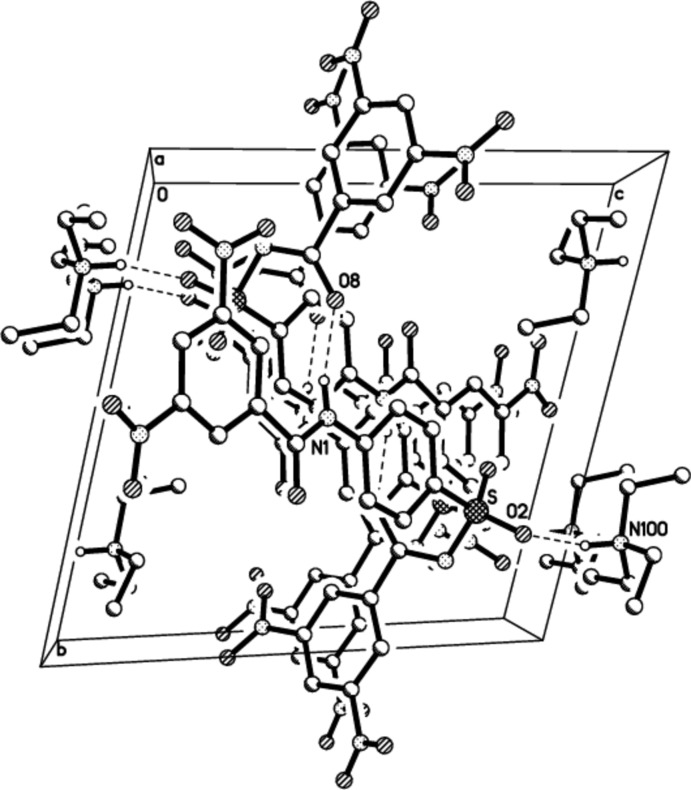
Packing diagram viewed along the a-axis showing N–H···O hydrogen bonding pattern as dashed lines. H-atoms not involved are omitted.

## supplementary crystallographic information

### Comment

In the past decade, the demand for polyamide-imide (PAI) and other high-temperature resistant polymeric materials has grown progressively because of their outstanding mechanical properties, excellent thermal stability (Kawakami *et al.*, 2003), etc. The title compound is a starting material for such types of materials.

### Experimental

In this preparation reagent grade quality chemicals were used without their further purification. In a 100 ml, three necked, round bottomed flask, equipped with a condenser, a nitrogen gas inlet tube, a thermometer and a magnetic stirrer, of sulfanilamide (1.39 g, 0.0086 mole) in dry dichloromethane (20 ml) and a few drops of N,*N*-dimethylformamide(DMF) stirred at 273–278 K for 30 minutes and 3,5- dinitrobenzoylchloride (3.72 g, 0.0161 mol) in dichloromethane (30 ml) was added dropwise by dropping funnel and stirring was continued for further 1 h under the same conditions. The reaction mixture was then refluxed for 45 min. The flask content was cooled to room temperature, poured into water and let it stand for 24 h. The resulting dark brown precipitates were filtered, washed with hot water and 5% NaOH solution. Finally, the product was washed with hot water and dried under vacuum at 350 K. The crude product was recrystallized from N,*N*-dimethylformamide(DMF). Yield: 87%; m.p 460–462 K.

### Refinement

Hydrogen atoms were clearly identified in difference syntheses, refined at idealized positions riding on the carbon or nitrogen atoms with C—H 0.95–0.98 Å, N—H 0.88 and 0.93 Å and with isotropic displacement parameters *U*_iso_(H) = 1.2U_eq_(C/N) or 1.5U_eq_(methyl C). All methyl hydrogen atoms were allowed to rotate but not to tip.

### Crystal data

**Table:**

C_6_H_16_N^+^·C_20_H_11_N_6_O_12_S^−^	*Z* = 2
*M**_r_* = 661.61	*F*(000) = 688
Triclinic, *P*1	*D*_x_ = 1.517 Mg m^−^^3^
Hall symbol: -P 1	Mo *K*α radiation, λ = 0.71073 Å
*a* = 9.1046 (12) Å	Cell parameters from 2907 reflections
*b* = 13.2050 (18) Å	θ = 2.4–26.9°
*c* = 13.3427 (18) Å	µ = 0.19 mm^−^^1^
α = 98.854 (3)°	*T* = 130 K
β = 105.147 (3)°	Prism, colourless
γ = 105.494 (3)°	0.38 × 0.37 × 0.19 mm
*V* = 1448.3 (3) Å^3^	

### Data collection

**Table:**

Bruker SMART APEX diffractometer	6875 independent reflections
Radiation source: sealed tube	4652 reflections with *I* > 2σ(*I*)
Graphite monochromator	*R*_int_ = 0.032
φ and ω scans	θ_max_ = 27.9°, θ_min_ = 1.6°
Absorption correction: multi-scan (*SADABS*; Sheldrick, 2004)	*h* = −11→10
*T*_min_ = 0.931, *T*_max_ = 0.965	*k* = −17→17
13859 measured reflections	*l* = −17→17

### Refinement

**Table:**

Refinement on *F*^2^	Primary atom site location: structure-invariant direct methods
Least-squares matrix: full	Secondary atom site location: difference Fourier map
*R*[*F*^2^ > 2σ(*F*^2^)] = 0.041	Hydrogen site location: difference Fourier map
*wR*(*F*^2^) = 0.084	H-atom parameters constrained
*S* = 0.89	*w* = 1/[σ^2^(*F*_o_^2^) + (0.029*P*)^2^] where *P* = (*F*_o_^2^ + 2*F*_c_^2^)/3
6875 reflections	(Δ/σ)_max_ = 0.001
415 parameters	Δρ_max_ = 0.36 e Å^−^^3^
0 restraints	Δρ_min_ = −0.36 e Å^−^^3^

### Special details

**Table:**

Geometry. All e.s.d.'s (except the e.s.d. in the dihedral angle between two l.s. planes) are estimated using the full covariance matrix. The cell e.s.d.'s are taken into account individually in the estimation of e.s.d.'s in distances, angles and torsion angles; correlations between e.s.d.'s in cell parameters are only used when they are defined by crystal symmetry. An approximate (isotropic) treatment of cell e.s.d.'s is used for estimating e.s.d.'s involving l.s. planes.
Refinement. Refinement of *F*^2^ against ALL reflections. The weighted *R*-factor *wR* and goodness of fit *S* are based on *F*^2^, conventional *R*-factors *R* are based on *F*, with *F* set to zero for negative *F*^2^. The threshold expression of *F*^2^ > σ(*F*^2^) is used only for calculating *R*-factors(gt) *etc*. and is not relevant to the choice of reflections for refinement. *R*-factors based on *F*^2^ are statistically about twice as large as those based on *F*, and *R*- factors based on ALL data will be even larger.

### Fractional atomic coordinates and isotropic or equivalent isotropic displacement parameters (Å^2^)

**Table:**

	*x*	*y*	*z*	*U*_iso_*/*U*_eq_	
S1	0.90395 (6)	0.28292 (3)	0.21593 (4)	0.01914 (11)	
O1	1.03295 (15)	0.36565 (9)	0.20308 (10)	0.0246 (3)	
O2	0.76199 (15)	0.23599 (9)	0.12131 (9)	0.0245 (3)	
O3	0.52517 (16)	0.33418 (9)	0.59254 (11)	0.0317 (3)	
O4	0.32249 (17)	0.35984 (10)	0.89035 (11)	0.0385 (4)	
O5	0.29330 (18)	0.51042 (11)	0.95560 (12)	0.0435 (4)	
O6	0.62662 (18)	0.84333 (10)	0.90660 (12)	0.0426 (4)	
O7	0.67579 (18)	0.83485 (10)	0.75614 (12)	0.0404 (4)	
O8	1.15593 (14)	0.29259 (9)	0.40479 (9)	0.0214 (3)	
O9	0.90468 (16)	−0.26946 (9)	0.35110 (11)	0.0317 (3)	
O10	0.73408 (15)	−0.18561 (9)	0.29762 (10)	0.0275 (3)	
O11	1.40360 (16)	−0.02522 (10)	0.63169 (10)	0.0315 (3)	
O12	1.53041 (14)	0.11153 (9)	0.58183 (10)	0.0248 (3)	
N1	0.69344 (17)	0.47991 (11)	0.56319 (11)	0.0187 (3)	
H1A	0.7360	0.5508	0.5846	0.022*	
N2	0.34552 (19)	0.45696 (13)	0.89879 (12)	0.0266 (4)	
N3	0.62909 (19)	0.79268 (12)	0.82266 (14)	0.0279 (4)	
N4	0.95176 (17)	0.18180 (11)	0.25028 (11)	0.0191 (3)	
N5	0.87053 (19)	−0.18774 (11)	0.33830 (12)	0.0214 (4)	
N6	1.40938 (18)	0.03787 (11)	0.57313 (12)	0.0208 (3)	
C1	0.8402 (2)	0.34117 (13)	0.31860 (13)	0.0169 (4)	
C2	0.8728 (2)	0.45227 (13)	0.34808 (13)	0.0188 (4)	
H2A	0.9308	0.4983	0.3136	0.023*	
C3	0.8206 (2)	0.49557 (13)	0.42770 (14)	0.0185 (4)	
H3A	0.8421	0.5716	0.4474	0.022*	
C4	0.7365 (2)	0.42858 (13)	0.47979 (14)	0.0173 (4)	
C5	0.7027 (2)	0.31665 (13)	0.44911 (14)	0.0201 (4)	
H5A	0.6452	0.2702	0.4836	0.024*	
C6	0.7535 (2)	0.27417 (13)	0.36838 (14)	0.0201 (4)	
H6A	0.7289	0.1980	0.3465	0.024*	
C7	0.5933 (2)	0.43160 (13)	0.61400 (14)	0.0189 (4)	
C8	0.5640 (2)	0.50400 (13)	0.70085 (14)	0.0176 (4)	
C9	0.4789 (2)	0.45188 (14)	0.76106 (14)	0.0193 (4)	
H9A	0.4461	0.3752	0.7488	0.023*	
C10	0.4427 (2)	0.51314 (14)	0.83887 (14)	0.0199 (4)	
C11	0.4892 (2)	0.62476 (14)	0.86136 (14)	0.0220 (4)	
H11A	0.4637	0.6657	0.9155	0.026*	
C12	0.5748 (2)	0.67311 (13)	0.80072 (15)	0.0209 (4)	
C13	0.6110 (2)	0.61631 (13)	0.72018 (14)	0.0198 (4)	
H13A	0.6670	0.6531	0.6786	0.024*	
C14	1.0699 (2)	0.20335 (13)	0.34280 (14)	0.0176 (4)	
C15	1.0969 (2)	0.10340 (13)	0.37607 (13)	0.0166 (4)	
C16	0.9761 (2)	0.00384 (13)	0.33703 (14)	0.0174 (4)	
H16A	0.8773	−0.0044	0.2848	0.021*	
C17	1.0032 (2)	−0.08325 (13)	0.37609 (14)	0.0168 (4)	
C18	1.1434 (2)	−0.07612 (13)	0.45135 (14)	0.0186 (4)	
H18A	1.1593	−0.1370	0.4768	0.022*	
C19	1.2601 (2)	0.02450 (13)	0.48797 (13)	0.0170 (4)	
C20	1.2409 (2)	0.11451 (13)	0.45224 (13)	0.0170 (4)	
H20A	1.3243	0.1823	0.4792	0.020*	
N100	0.22811 (19)	0.79060 (12)	0.08484 (12)	0.0261 (4)	
H10P	0.2155	0.7935	0.0140	0.031*	
C101	0.1711 (3)	0.87782 (16)	0.13073 (18)	0.0390 (6)	
H10A	0.2443	0.9491	0.1326	0.047*	
H10B	0.1762	0.8753	0.2053	0.047*	
C102	0.0024 (3)	0.8674 (2)	0.0680 (2)	0.0595 (7)	
H10C	−0.0289	0.9261	0.1014	0.089*	
H10D	−0.0711	0.7976	0.0670	0.089*	
H10E	−0.0029	0.8717	−0.0055	0.089*	
C103	0.1294 (3)	0.67768 (15)	0.08118 (17)	0.0356 (5)	
H10F	0.0170	0.6640	0.0370	0.043*	
H10G	0.1708	0.6253	0.0455	0.043*	
C104	0.1301 (3)	0.65733 (18)	0.18919 (19)	0.0483 (6)	
H10H	0.0635	0.5827	0.1804	0.072*	
H10I	0.0867	0.7076	0.2246	0.072*	
H10J	0.2404	0.6685	0.2329	0.072*	
C105	0.4047 (2)	0.81232 (17)	0.13686 (16)	0.0355 (5)	
H10K	0.4334	0.7480	0.1117	0.043*	
H10L	0.4280	0.8243	0.2153	0.043*	
C106	0.5073 (3)	0.91023 (18)	0.11218 (17)	0.0456 (6)	
H10M	0.6212	0.9213	0.1476	0.068*	
H10N	0.4809	0.9744	0.1383	0.068*	
H10O	0.4863	0.8982	0.0347	0.068*	

### Atomic displacement parameters (Å^2^)

**Table:**

	*U*^11^	*U*^22^	*U*^33^	*U*^12^	*U*^13^	*U*^23^
S1	0.0226 (3)	0.0190 (2)	0.0165 (2)	0.00746 (18)	0.0059 (2)	0.00580 (18)
O1	0.0280 (8)	0.0239 (7)	0.0267 (8)	0.0091 (6)	0.0123 (6)	0.0122 (6)
O2	0.0251 (8)	0.0297 (7)	0.0161 (7)	0.0102 (6)	0.0017 (6)	0.0040 (5)
O3	0.0382 (9)	0.0167 (7)	0.0389 (9)	0.0000 (6)	0.0229 (7)	0.0002 (6)
O4	0.0561 (10)	0.0260 (8)	0.0365 (9)	0.0065 (7)	0.0256 (8)	0.0098 (6)
O5	0.0544 (11)	0.0416 (9)	0.0526 (10)	0.0203 (8)	0.0401 (9)	0.0142 (8)
O6	0.0566 (11)	0.0248 (8)	0.0472 (10)	0.0089 (7)	0.0299 (8)	−0.0048 (7)
O7	0.0565 (10)	0.0213 (7)	0.0529 (10)	0.0103 (7)	0.0336 (9)	0.0116 (7)
O8	0.0245 (7)	0.0144 (6)	0.0221 (7)	0.0037 (5)	0.0052 (6)	0.0039 (5)
O9	0.0339 (8)	0.0145 (7)	0.0436 (9)	0.0064 (6)	0.0071 (7)	0.0098 (6)
O10	0.0194 (8)	0.0227 (7)	0.0343 (8)	0.0022 (6)	0.0048 (6)	0.0043 (6)
O11	0.0352 (8)	0.0266 (7)	0.0286 (8)	0.0061 (6)	0.0020 (7)	0.0155 (6)
O12	0.0196 (7)	0.0195 (7)	0.0305 (8)	0.0026 (5)	0.0051 (6)	0.0044 (6)
N1	0.0228 (9)	0.0109 (7)	0.0210 (8)	0.0035 (6)	0.0074 (7)	0.0021 (6)
N2	0.0263 (10)	0.0307 (9)	0.0228 (9)	0.0073 (7)	0.0088 (8)	0.0081 (7)
N3	0.0250 (10)	0.0209 (8)	0.0377 (11)	0.0072 (7)	0.0131 (8)	0.0020 (8)
N4	0.0223 (9)	0.0173 (7)	0.0180 (8)	0.0080 (6)	0.0051 (7)	0.0045 (6)
N5	0.0262 (10)	0.0159 (8)	0.0207 (9)	0.0032 (7)	0.0096 (7)	0.0034 (6)
N6	0.0239 (9)	0.0171 (8)	0.0210 (9)	0.0072 (6)	0.0064 (7)	0.0037 (6)
C1	0.0172 (10)	0.0182 (9)	0.0136 (9)	0.0068 (7)	0.0010 (8)	0.0036 (7)
C2	0.0200 (10)	0.0185 (9)	0.0169 (10)	0.0047 (7)	0.0041 (8)	0.0069 (7)
C3	0.0197 (10)	0.0128 (8)	0.0204 (10)	0.0038 (7)	0.0030 (8)	0.0048 (7)
C4	0.0150 (9)	0.0178 (9)	0.0168 (10)	0.0054 (7)	0.0018 (8)	0.0037 (7)
C5	0.0209 (10)	0.0171 (9)	0.0232 (10)	0.0051 (7)	0.0083 (8)	0.0068 (8)
C6	0.0232 (10)	0.0143 (9)	0.0222 (10)	0.0063 (7)	0.0057 (8)	0.0049 (7)
C7	0.0167 (10)	0.0169 (9)	0.0208 (10)	0.0042 (7)	0.0039 (8)	0.0039 (7)
C8	0.0147 (9)	0.0175 (9)	0.0180 (10)	0.0049 (7)	0.0022 (8)	0.0029 (7)
C9	0.0168 (10)	0.0178 (9)	0.0205 (10)	0.0042 (7)	0.0024 (8)	0.0045 (7)
C10	0.0156 (10)	0.0251 (10)	0.0179 (10)	0.0050 (7)	0.0045 (8)	0.0067 (8)
C11	0.0201 (10)	0.0239 (10)	0.0213 (10)	0.0084 (8)	0.0058 (8)	0.0027 (8)
C12	0.0181 (10)	0.0170 (9)	0.0260 (11)	0.0064 (7)	0.0048 (8)	0.0032 (8)
C13	0.0176 (10)	0.0184 (9)	0.0232 (10)	0.0047 (7)	0.0073 (8)	0.0052 (8)
C14	0.0178 (10)	0.0161 (9)	0.0220 (10)	0.0051 (7)	0.0108 (8)	0.0056 (7)
C15	0.0205 (10)	0.0163 (9)	0.0151 (9)	0.0058 (7)	0.0091 (8)	0.0041 (7)
C16	0.0186 (10)	0.0182 (9)	0.0171 (9)	0.0070 (7)	0.0078 (8)	0.0035 (7)
C17	0.0200 (10)	0.0134 (8)	0.0165 (10)	0.0031 (7)	0.0089 (8)	0.0009 (7)
C18	0.0254 (11)	0.0153 (9)	0.0193 (10)	0.0081 (7)	0.0114 (8)	0.0059 (7)
C19	0.0171 (10)	0.0199 (9)	0.0149 (9)	0.0067 (7)	0.0055 (8)	0.0045 (7)
C20	0.0206 (10)	0.0153 (8)	0.0164 (10)	0.0046 (7)	0.0096 (8)	0.0030 (7)
N100	0.0316 (10)	0.0294 (9)	0.0178 (9)	0.0109 (7)	0.0082 (8)	0.0051 (7)
C101	0.0514 (15)	0.0340 (12)	0.0410 (14)	0.0219 (11)	0.0225 (12)	0.0080 (10)
C102	0.0512 (18)	0.0747 (19)	0.076 (2)	0.0408 (15)	0.0293 (15)	0.0307 (15)
C103	0.0432 (14)	0.0290 (11)	0.0350 (13)	0.0087 (10)	0.0163 (11)	0.0073 (9)
C104	0.0536 (16)	0.0516 (15)	0.0494 (16)	0.0169 (12)	0.0232 (13)	0.0281 (12)
C105	0.0306 (13)	0.0507 (14)	0.0224 (12)	0.0139 (10)	0.0034 (10)	0.0080 (10)
C106	0.0334 (14)	0.0634 (16)	0.0289 (13)	0.0030 (11)	0.0069 (11)	0.0072 (11)

### Geometric parameters (Å, º)

**Table:**

S1—O1	1.4421 (12)	C10—C11	1.381 (2)
S1—O2	1.4584 (12)	C11—C12	1.376 (2)
S1—N4	1.6053 (14)	C11—H11A	0.9500
S1—C1	1.7708 (18)	C12—C13	1.377 (2)
O3—C7	1.2203 (19)	C13—H13A	0.9500
O4—N2	1.2251 (18)	C14—C15	1.515 (2)
O5—N2	1.2255 (19)	C15—C16	1.388 (2)
O6—N3	1.2218 (19)	C15—C20	1.389 (2)
O7—N3	1.2253 (19)	C16—C17	1.387 (2)
O8—C14	1.2468 (19)	C16—H16A	0.9500
O9—N5	1.2242 (18)	C17—C18	1.373 (2)
O10—N5	1.2288 (18)	C18—C19	1.381 (2)
O11—N6	1.2272 (17)	C18—H18A	0.9500
O12—N6	1.2245 (17)	C19—C20	1.383 (2)
N1—C7	1.358 (2)	C20—H20A	0.9500
N1—C4	1.410 (2)	N100—C101	1.496 (2)
N1—H1A	0.8800	N100—C105	1.502 (2)
N2—C10	1.474 (2)	N100—C103	1.507 (2)
N3—C12	1.479 (2)	N100—H10P	0.9300
N4—C14	1.340 (2)	C101—C102	1.503 (3)
N5—C17	1.481 (2)	C101—H10A	0.9900
N6—C19	1.474 (2)	C101—H10B	0.9900
C1—C2	1.387 (2)	C102—H10C	0.9800
C1—C6	1.388 (2)	C102—H10D	0.9800
C2—C3	1.379 (2)	C102—H10E	0.9800
C2—H2A	0.9500	C103—C104	1.505 (3)
C3—C4	1.399 (2)	C103—H10F	0.9900
C3—H3A	0.9500	C103—H10G	0.9900
C4—C5	1.397 (2)	C104—H10H	0.9800
C5—C6	1.379 (2)	C104—H10I	0.9800
C5—H5A	0.9500	C104—H10J	0.9800
C6—H6A	0.9500	C105—C106	1.517 (3)
C7—C8	1.511 (2)	C105—H10K	0.9900
C8—C9	1.389 (2)	C105—H10L	0.9900
C8—C13	1.391 (2)	C106—H10M	0.9800
C9—C10	1.380 (2)	C106—H10N	0.9800
C9—H9A	0.9500	C106—H10O	0.9800
			
O1—S1—O2	114.78 (8)	O8—C14—C15	116.86 (16)
O1—S1—N4	114.71 (8)	N4—C14—C15	113.96 (14)
O2—S1—N4	105.36 (7)	C16—C15—C20	120.30 (15)
O1—S1—C1	108.37 (8)	C16—C15—C14	120.81 (16)
O2—S1—C1	106.53 (8)	C20—C15—C14	118.73 (15)
N4—S1—C1	106.51 (8)	C17—C16—C15	118.37 (17)
C7—N1—C4	126.94 (14)	C17—C16—H16A	120.8
C7—N1—H1A	116.5	C15—C16—H16A	120.8
C4—N1—H1A	116.5	C18—C17—C16	123.38 (16)
O4—N2—O5	123.33 (16)	C18—C17—N5	118.55 (15)
O4—N2—C10	118.61 (15)	C16—C17—N5	118.00 (16)
O5—N2—C10	118.05 (15)	C17—C18—C19	116.22 (16)
O6—N3—O7	124.04 (16)	C17—C18—H18A	121.9
O6—N3—C12	118.00 (16)	C19—C18—H18A	121.9
O7—N3—C12	117.95 (15)	C18—C19—C20	123.31 (17)
C14—N4—S1	116.77 (12)	C18—C19—N6	118.34 (15)
O9—N5—O10	124.93 (14)	C20—C19—N6	118.28 (15)
O9—N5—C17	117.63 (16)	C19—C20—C15	118.41 (15)
O10—N5—C17	117.43 (14)	C19—C20—H20A	120.8
O12—N6—O11	124.18 (16)	C15—C20—H20A	120.8
O12—N6—C19	118.27 (14)	C101—N100—C105	112.12 (15)
O11—N6—C19	117.55 (14)	C101—N100—C103	114.20 (16)
C2—C1—C6	119.86 (16)	C105—N100—C103	112.11 (15)
C2—C1—S1	120.83 (13)	C101—N100—H10P	105.9
C6—C1—S1	119.30 (13)	C105—N100—H10P	105.9
C3—C2—C1	119.70 (16)	C103—N100—H10P	105.9
C3—C2—H2A	120.2	N100—C101—C102	112.84 (18)
C1—C2—H2A	120.2	N100—C101—H10A	109.0
C2—C3—C4	120.64 (16)	C102—C101—H10A	109.0
C2—C3—H3A	119.7	N100—C101—H10B	109.0
C4—C3—H3A	119.7	C102—C101—H10B	109.0
C5—C4—C3	119.39 (16)	H10A—C101—H10B	107.8
C5—C4—N1	123.68 (15)	C101—C102—H10C	109.5
C3—C4—N1	116.91 (15)	C101—C102—H10D	109.5
C6—C5—C4	119.44 (16)	H10C—C102—H10D	109.5
C6—C5—H5A	120.3	C101—C102—H10E	109.5
C4—C5—H5A	120.3	H10C—C102—H10E	109.5
C5—C6—C1	120.93 (16)	H10D—C102—H10E	109.5
C5—C6—H6A	119.5	C104—C103—N100	114.20 (17)
C1—C6—H6A	119.5	C104—C103—H10F	108.7
O3—C7—N1	123.77 (16)	N100—C103—H10F	108.7
O3—C7—C8	118.86 (16)	C104—C103—H10G	108.7
N1—C7—C8	117.37 (14)	N100—C103—H10G	108.7
C9—C8—C13	119.55 (16)	H10F—C103—H10G	107.6
C9—C8—C7	116.10 (15)	C103—C104—H10H	109.5
C13—C8—C7	124.31 (16)	C103—C104—H10I	109.5
C10—C9—C8	119.00 (16)	H10H—C104—H10I	109.5
C10—C9—H9A	120.5	C103—C104—H10J	109.5
C8—C9—H9A	120.5	H10H—C104—H10J	109.5
C9—C10—C11	123.09 (16)	H10I—C104—H10J	109.5
C9—C10—N2	118.65 (15)	N100—C105—C106	112.26 (17)
C11—C10—N2	118.23 (16)	N100—C105—H10K	109.2
C12—C11—C10	115.99 (16)	C106—C105—H10K	109.2
C12—C11—H11A	122.0	N100—C105—H10L	109.2
C10—C11—H11A	122.0	C106—C105—H10L	109.2
C11—C12—C13	123.60 (16)	H10K—C105—H10L	107.9
C11—C12—N3	118.04 (16)	C105—C106—H10M	109.5
C13—C12—N3	118.36 (16)	C105—C106—H10N	109.5
C12—C13—C8	118.73 (16)	H10M—C106—H10N	109.5
C12—C13—H13A	120.6	C105—C106—H10O	109.5
C8—C13—H13A	120.6	H10M—C106—H10O	109.5
O8—C14—N4	129.16 (16)	H10N—C106—H10O	109.5
			
O1—S1—N4—C14	59.63 (15)	O7—N3—C12—C11	−165.52 (17)
O2—S1—N4—C14	−173.15 (13)	O6—N3—C12—C13	−165.24 (17)
C1—S1—N4—C14	−60.25 (14)	O7—N3—C12—C13	13.8 (2)
O1—S1—C1—C2	20.61 (16)	C11—C12—C13—C8	−2.4 (3)
O2—S1—C1—C2	−103.39 (14)	N3—C12—C13—C8	178.36 (15)
N4—S1—C1—C2	144.52 (14)	C9—C8—C13—C12	1.5 (3)
O1—S1—C1—C6	−160.73 (13)	C7—C8—C13—C12	178.89 (16)
O2—S1—C1—C6	75.26 (15)	S1—N4—C14—O8	−4.3 (3)
N4—S1—C1—C6	−36.83 (16)	S1—N4—C14—C15	173.89 (11)
C6—C1—C2—C3	0.8 (2)	O8—C14—C15—C16	156.24 (16)
S1—C1—C2—C3	179.47 (14)	N4—C14—C15—C16	−22.2 (2)
C1—C2—C3—C4	0.6 (3)	O8—C14—C15—C20	−19.3 (2)
C2—C3—C4—C5	−1.2 (3)	N4—C14—C15—C20	162.27 (15)
C2—C3—C4—N1	177.44 (16)	C20—C15—C16—C17	0.1 (2)
C7—N1—C4—C5	−11.5 (3)	C14—C15—C16—C17	−175.36 (15)
C7—N1—C4—C3	169.93 (16)	C15—C16—C17—C18	−0.1 (3)
C3—C4—C5—C6	0.3 (3)	C15—C16—C17—N5	176.81 (14)
N1—C4—C5—C6	−178.26 (16)	O9—N5—C17—C18	−20.4 (2)
C4—C5—C6—C1	1.2 (3)	O10—N5—C17—C18	158.70 (16)
C2—C1—C6—C5	−1.7 (3)	O9—N5—C17—C16	162.53 (15)
S1—C1—C6—C5	179.59 (14)	O10—N5—C17—C16	−18.3 (2)
C4—N1—C7—O3	−0.3 (3)	C16—C17—C18—C19	0.2 (3)
C4—N1—C7—C8	−179.45 (15)	N5—C17—C18—C19	−176.71 (15)
O3—C7—C8—C9	9.1 (2)	C17—C18—C19—C20	−0.3 (3)
N1—C7—C8—C9	−171.69 (15)	C17—C18—C19—N6	176.70 (15)
O3—C7—C8—C13	−168.29 (17)	O12—N6—C19—C18	156.51 (16)
N1—C7—C8—C13	10.9 (3)	O11—N6—C19—C18	−24.3 (2)
C13—C8—C9—C10	0.1 (3)	O12—N6—C19—C20	−26.3 (2)
C7—C8—C9—C10	−177.50 (16)	O11—N6—C19—C20	152.85 (16)
C8—C9—C10—C11	−1.0 (3)	C18—C19—C20—C15	0.3 (3)
C8—C9—C10—N2	177.07 (15)	N6—C19—C20—C15	−176.66 (15)
O4—N2—C10—C9	11.8 (2)	C16—C15—C20—C19	−0.2 (2)
O5—N2—C10—C9	−169.00 (17)	C14—C15—C20—C19	175.33 (15)
O4—N2—C10—C11	−170.03 (16)	C105—N100—C101—C102	170.41 (18)
O5—N2—C10—C11	9.2 (2)	C103—N100—C101—C102	−60.7 (2)
C9—C10—C11—C12	0.3 (3)	C101—N100—C103—C104	−62.2 (2)
N2—C10—C11—C12	−177.79 (16)	C105—N100—C103—C104	66.7 (2)
C10—C11—C12—C13	1.4 (3)	C101—N100—C105—C106	−67.0 (2)
C10—C11—C12—N3	−179.29 (15)	C103—N100—C105—C106	163.00 (17)
O6—N3—C12—C11	15.4 (2)		

### Hydrogen-bond geometry (Å, º)

**Table:**

*D*—H···*A*	*D*—H	H···*A*	*D*···*A*	*D*—H···*A*
N1—H1*A*···O8^i^	0.88	2.00	2.8611 (17)	165
N100—H10*P*···O2^ii^	0.93	1.86	2.747 (2)	158
C13—H13*A*···O8^i^	0.95	2.22	3.136 (2)	162
C3—H3*A*···O8^i^	0.95	2.45	3.221 (2)	139
C6—H6*A*···O11^iii^	0.95	2.38	3.215 (2)	147
C9—H9*A*···O10^iv^	0.95	2.48	3.384 (2)	159
C20—H20*A*···O3^v^	0.95	2.31	3.225 (2)	163

Symmetry codes: (i) −*x*+2, −*y*+1, −*z*+1; (ii) −*x*+1, −*y*+1, −*z*; (iii) −*x*+2, −*y*, −*z*+1; (iv) −*x*+1, −*y*, −*z*+1; (v) *x*+1, *y*, *z*.
